# Age estimation for two Mediterranean populations: rib histomorphometry applied to forensic identification and bone remodelling research

**DOI:** 10.1007/s00414-022-02812-2

**Published:** 2022-04-08

**Authors:** Julieta G. García-Donas, Robert R. Paine, Andrea Bonicelli, Elena F. Kranioti

**Affiliations:** 1 Center for Anatomy and Human Identification, School of Science and Engineering, University of Dundee, Dundee, UK; 2 Edinburgh Unit for Forensic Anthropology, School of History Classics and Archaeology, University of Edinburgh, Edinburgh, UK; 3 Department of Environmental Biology, Sapienza” University of Rome, P.le Aldo Moro 5, Rome, Italy; 4 Forensic Science Research Group, Faculty of Health and Life Sciences, Applied Sciences, Northumbria University, Newcastle Upon Tyne, NE1 8ST UK; 5 Forensic Medicine Unit, Department of Forensic Sciences, School of Medicine, University of Crete, Heraklion, Crete, Greece

**Keywords:** Rib, Cortical remodelling, Age estimation, Mediterranean samples, Forensic identification

## Abstract

Numerous intrinsic and extrinsic factors influence bone remodelling rates and have shown to affect the accuracy of histological aging methods. The present study investigates the rib cortex from two Mediterranean skeletal collections exploring the development of population-specific standards for histomorphometric age-at-death estimation. Eighty-eight standard ribs from two samples, Cretans and Greek-Cypriots, were processed histologically. Thirteen raw and composite histomorphometric parameters were assessed and observer error tested. The correlation between age and the parameters and the differences between sex and population subsamples were explored through group comparisons and analysis of covariance. General linear models assessed through data fit indicators and cross-validation were generated from the total dataset, and by sex and population subsamples. Most of the histological variables showed a statistically significant correlation with age with some differences observed by sex and by sample. From the twelve models generated, the optimal model for the whole sample included osteon population density (OPD), osteon perimeter, and osteon circularity producing an error of 10.71 years. When sex and samples were separated, the best model selected included OPD and osteon perimeter producing an error of 8.07 years for Greek-Cypriots. This research demonstrates the feasibility of quantitative bone histology to estimate age, obtaining errors rates in accordance with macroscopic ageing techniques. Sex and sample population differences need further investigation and inter-population variation in remodelling rates is suggested. Moreover, this study contributes to the creation of population-specific standards for Cretans and Greek-Cypriots.

**Supplementary Information:**

The online version contains supplementary material available at 10.1007/s00414-022-02812-2.

## Introduction

Micro-anatomical features of bone used in forensic age estimation methods were first developed using long bones such as femur, tibia, and fibula [1]. Nearly 30 years later, non-weight bearing bones such as the rib and clavicle were also used for age-at-death (ADD) estimation [2]. Since then, costal elements have been extensively used for histological age estimation [3, 4].

A variety of bone histological features (e.g. secondary osteons number, osteon metrics) applied on age estimation methods have demonstrated different correlation to age [3–5]. Moreover, the accuracy of the age estimates obtained have shown to be further dependent on intrinsic and extrinsic factors such as historic versus modern samples, pathology and physical activity [6, 7]. Additionally, methodological approaches and observer’s experience can also influence the results [8, 9].

Controversial conclusions have been drawn regarding inter-population variation age-related changes observed in bone microstructure [3, 10, 11]. Some studies have demonstrated that sample demographics and intrinsic sample characteristics do not have an impact on the reliability of the methods, reporting similar accuracy rates for age estimation population-specific methods as for non-related population formulae [10]. Other researchers have shown that the application of histological population-specific methods provides more accurate age estimates as existing methods produced age estimates not falling within the reported error rates [11–13]. Thus, exploring the development of population-specific histological age estimation formulae has been the target of many studies [4, 14].

This paper is a continuation of previously reported results specific to the validation study performed by testing four existing rib histological methods on Cretan and Greek-Cypriot samples [12]. A systematic underestimation of three of the aging methods with an overall increase in errors as age increases has been demonstrated [2, 3, 15]. Furthermore, higher accuracy of one of the methods was noticed although just for individuals over 60 years of age [5]. In view of these findings, rib histomorphometric parameters are further investigated in the current study to understand the implications of bone remodelling and age-related changes observed on rib cortical bone on the Mediterranean sample (Cretan and Greek-Cypriots). Accordingly, this paper proposes revised population-specific standard methods to estimate AAD through rib histomorphometry for Modern Mediterranean populations.

## Materials and methods

### Materials

**Fig. 1:**
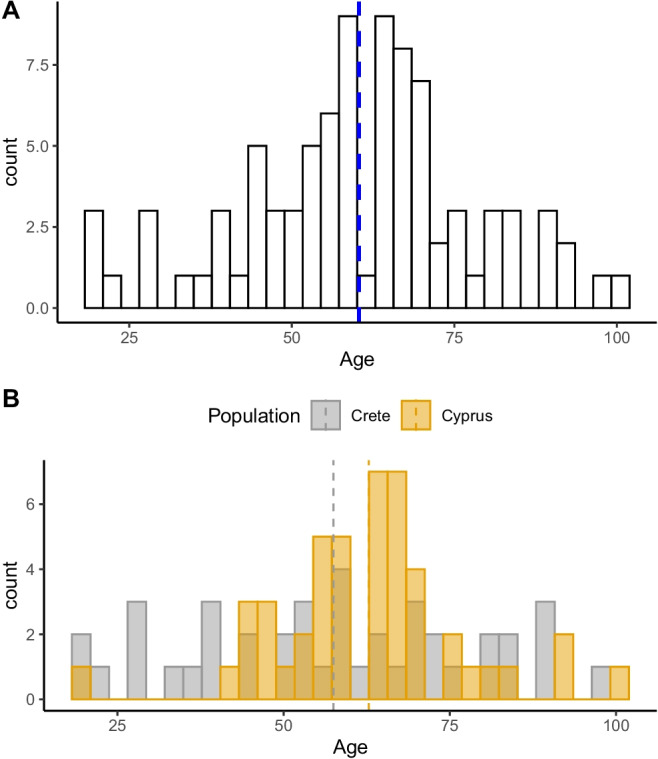
Age distribution of the total population (**A**) and the sample divided by populations (Crete and Greek-Cypriots) (**B**). Dashed line represents mean for each sample cluster

Two Mediterranean populations of known AAD and sex were used for this study (Table 1) [12, 16]. Ethical approval for this research was given by the University Hospital of Heraklion (Crete) and the University of Edinburgh (UK) ethics committees. Cretan sample consisted of 41 individuals, 34 individuals from the Cretan Osteological collection [17], and seven individuals collected from routine autopsy examination at the Forensic Medicine Unit of the University of Crete. A total of 41 individuals (23 males and 18 females) with a mean age of 57.48 (SD = 21.17) were included. The Greek-Cypriot sample was collected from individuals from the Cypriot Osteological Collection (Limassol, Republic of Cyprus) [18]. A total of 47 individuals (17 males and 30 females) with a mean age of 62.81 (SD = 14.20) were included. The combination of both samples resulted in a total number of 88 individuals with a mean age of 60.33 (SD = 17.89) (Table 1). The age distribution of the total sample as well as the sample divided by populations can be seen in Fig. 1.

**Table 1:** Demographic population data for the populations under study and the total sample

		*N*	Age range	Mean age	SD
Cretan sample (1968–2014)*	Males	23	19–89	55.54	20.5
	Females	18	27–98	60.23	22.42
	*Total*	*41*	*19–98*	*57.48*	*21.17*
Greek-Cypriot sample (1976–2003)*	Males	17	42–84	64.11	10.9
	Females	30	45–100	62.06	15.9
	*Total*	*47*	*20–100*	*62.81*	*14.2*
Pooled sample	Males	40	20–89	60.10	16.53
	Females	48	19–100	60.52	19.11
	*Total*	*88*	*19–100*	*60.33*	*17.89*

*Year of death for individuals in the sample

### Methods

The target costal element for this study is the 6th left rib; however, left or right standard ribs (4th–8th) were collected when the 6th rib was not available, as no bias is reported by the histomorphometric examination of other standard ribs [19]. The sampling area selected was the rib midshaft with the inclusion criteria considering no damage of the periosteal surface and no trauma or evident pathology affecting bone metabolism.

**Fig. 2:**
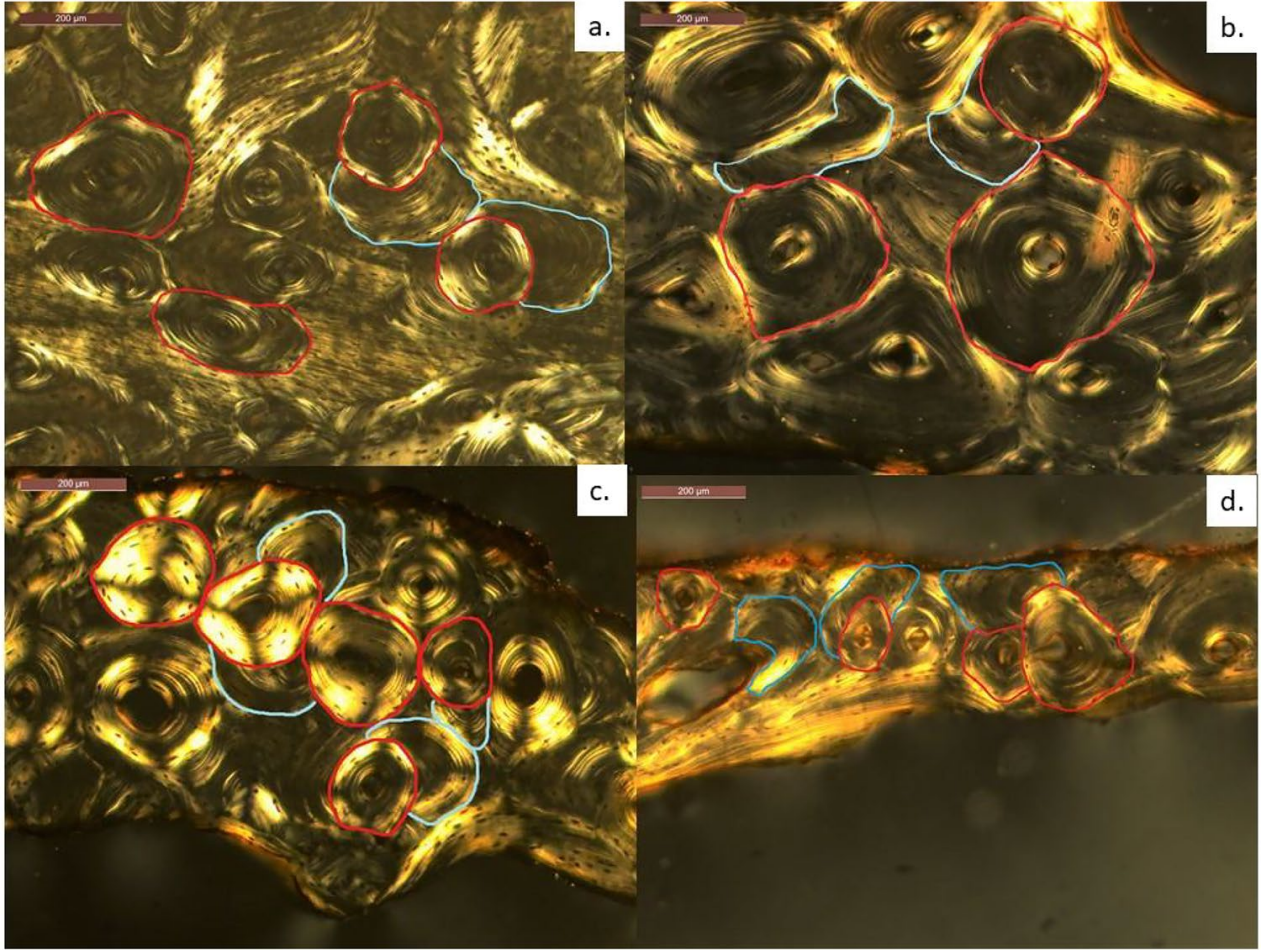
Cortical bone microstructure observed in different individuals: **a** 20 years old, **b** 51 years old, **c** 70 years old, **d** 91 years old. Red outline: examples of intact secondary osteons; blue outline: examples of fragmentary secondary osteons

Thin-sections were processed following standard procedures for bone histological analysis [20, 21]. A Leica DM 750P research microscope fitted with a Leica MC 170 HD camera (Leica Application Suite V4 software) was used for capturing 4 × and 10 × magnification microphotographs employed for data collection. Table 2 includes the definition and related data acquisition procedure for all raw and composite histomorphometric parameters explored in this study. Cross-section area measurements were taken on stitched microphotographs, and osteon counting was performed using a standard research light microscope (with polarised filter). The microphotographs were also used to record a count of micro-structures. Single osteon metric parameters were collected through 10 × magnification semi-polarised microphotographs using the segmented tool available in Image J 1.48 software platform [22]. Examples of different age cohort individuals with examples of the parameters collected are presented in Fig. 2.

**Table 2:** Raw and composite histomorphometric parameters assessed

Variable	Abbreviation	Brief definition	Author	Calculation	Data acquisition
Intact osteon number	N.On	Secondary osteon number with 90% of the Haversian canal perimeter showing no evidence of resorption	Stout and Paine [1992] [2]	n/a	Microscopy and microphotographs (histomorphology qualitative observation)
Fragmentary osteon number	N.On.Fg	Secondary osteon number with 10% or more of the Haversian canal perimeter showing evidence of resorption		n/a	
Total osteons	N.On.Tt	Sum of intact osteons and fragmentary osteons		N.On + N.On.Fg	
Intact osteon density	OPD(I)	Intact osteon number divided by cortical area (#/mm^2^)		N.On/Ct. Ar	
Fragmentary osteon density	OPD(F)	Fragmentary osteon number divided by cortical area (#/mm^2^)		N.On.Fg/Ct.Ar	
Total visible osteon density	OPD	Sum of Intact osteons and Fragmentary osteons divided by cortical area (#/mm^2^)		N.On + N.On.Fg/Ct.Ar	
Cortical area	Ct.Ar	Cortical area sampled (mm^2^)	Cho et al. [2006] [3]	Tt.Ar-Es.Ar	ImageJ software
Total area	Tt.Ar	Surface area including cortical and trabecular areas (mm^2^)		n/a	
Endosteal area	Es.Ar	Area occupied by trabecular bone (mm^2^)		n/a	
Relative cortical area	Ct.Ar/Tt.Ar	Ratio of cortical area to total area		Ct.Ar./Tt.Ar	
Osteon area	On.Ar	Area within the cement line of an intact secondary osteon (mm^2^)	Cho et al. [2002] [3]	n/a	
Osteon perimeter	On.Pm	Perimeter of the area within the cement line of an intact secondary osteon (mm^2^)	Thompson and Galvin [1983] [23]	n/a	
Osteon circularity	On.Cr	Measure of the proximity of an osteon to a true circle (index)	Goliath et al. [2016] [5]	(4π (area/perimeter^2^)	

*n/a* not applicable.

Intra- and inter-observer errors have been already reported for the present histological data [12]. Considering both Technical error of measurement (TEM) and Intra-class Correlation Coefficient (ICC) results, it was demonstrated that overall within and between observer errors fell within the acceptable levels of error, except for N.On.Fg, osteon population density(F) (OPD(F)) and On.Cr that showed that 15–30% of the variance can be attributed to measurement error and moderate to good agreement was reported.

Osteon perimeter observer errors were not tested previously, and the results will be reported here. A total of 22 thin sections were randomly selected from the total sample with intra-observer error being performed on the slides after 3 months from first data collection, while inter-observer was performed by one of the co-authors who has basic training in histomorphometric analysis. Observer error was assessed through TEM reporting TEM values, relative TEM (rTEM), and R as well as through ICC [24, 25]. ICC was performed using a two-way mixed effects model under absolute agreement with over 0.80 and 0.90 ICC and 95% confidence intervals considered as good and excellent agreement, respectively [26]. The histomorphometric data was assessed and analyzed through different statistical tests. First, descriptive statistics were calculated to present simple descriptors. Normal distribution of the data was explored through histograms, Q-Q plots, and Shapiro–Wilk test [27]. Depending on the distribution of the data, the relationship between the parameters and age for the total sample was assessed using Pearson’s correlation or Spearman’s Rank correlation coefficients [28]. The age distribution between sex groups and populations was compared using Welch’s *t*-test [29]. Depending on whether the assumption of normality was confirmed or not, Welch´s *t*-test or Mann–Whitney *U* were performed to compare the differences on the histomorphometric parameters and age in the two groups, sex and population samples, separately. One-way analysis of covariance (ANCOVA) was performed to further explore the relationship between age, samples (sex and population) and the parameters. The dependent variable was each histological parameter, and group was set as the factor (either sex or population sample). This analysis allowed to determine whether group membership had a significant effect on the dependent variables and whether age was a significant covariate [30]. If the variables were not normally distributed, the natural log transformation was performed. Finally, general linear regression models (GLM) were generated through linear and multiple regression analyses to produce the age estimation equations for the total sample and sub-samples (sex and population, separately). Residuals were inspected for the assumptions of linearity, normality, independence, and homogeneity of variance [31, 32]. For multiple linear regression analysis, independence of the parameters was examined through bivariate correlation, tolerance, and the variance inflation factor statistics (VIF). The GLM models were performed using Gaussian link function and maximum likelihood fitting assessing model selection according to significance levels, ANOVA *x*^2^ test as well as Akaike information criterion (AIC) and Bayesian information criterion (BIC) [33]. Moreover, goodness of fit indicators such as *R*^2^ and standard error of the estimate (SEE) were considered as well as the number, repeatability, and reproducibility of the parameters for the final selection of the optimal models. Leave-one-out (LOO) cross-validation was performed for all regression models to avoid splitting the sample (https://github.com/topepo/caret). Cross-validated results were compared to the initial regression results by means of adjusted *R*^2^, cross-validated SEE, and mean absolute error (MAE). Statistical analysis was performed using IBM SPSS 27 and R version 4.1.0.

## Results

### Intra- and inter-observer error-On.Pm

On.Pm intra- and inter-observer error values as obtained through TEM analysis fell within the limits of agreement with rTEM and R reporting values under 5 and above 0.95, respectively. ICC analysis reports excellent agreement for both intra- and inter-observer error with ICC estimates over 0.98 and 95% confidence intervals falling between 0.96 and 0.99 indicating excellent agreement.

### Histomorphometric parameters and age

**Fig. 3:**
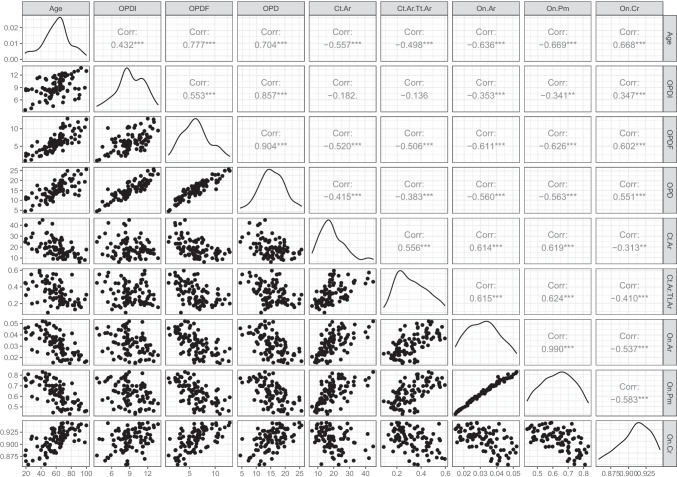
Age and the histomorphometric parameters for the entire rib sample presenting correlation coefficients, scatterplot matrix, and density plots showing the distribution of the variables across the sample. **p-value < 0.01, ***p-value < 0.001

Table 3 presents data for the total sample including descriptive statistics for the raw and composite histomorphometric parameters. Pearson’s correlation coefficient or Spearman’s rank correlation coefficient for each parameter are reported (*see supplementary material for sex and population subgroups*). Among all the variables, the highest correlation with age was reported for OPD(F), OPD, On.Pm, and On.Cr. Figure 3 presents information about the relationship between age and the parameters. Density plots show the frequency distribution of each parameter. The relationship between the parameters is presented using Pearson’s correlation coefficients. The scatter plot matrices not only show the relationship between each parameter and age, but also the relationship between each parameter. For example, it can be seen that osteon area and osteon perimeter have a strong positive correlation. Note that only Pearson’s correlation coefficients are reported with the difference between Pearson’s and Spearman’s values being minimal considering sample size effect.

**Table 3:** Descriptive statistics and correlation coefficient for raw and composite histomorphometric parameters for the total sample

Variables (*N* = 88)	Minimum	Maximum	Mean	SD	r/*rho*
Known age	19	100	60.33	17.89	N/A
N.On	46	399	173	73.49	− 0.23*
N.On.Fg	23	224	110	45.9	0.31***
N.On.Tt	96	583	282	107.81	− 0.02
OPD(I)	3.56	13.72	9.16	2.23	0.43***
OPD(F)	0.93	12.85	6.28	2.69	0.78***
OPD	4.49	25.62	15.44	4.35	0.71***
Ct.Ar	6.38	44.77	19.21	7.98	− 0.58***
Tt.Ar	26.82	155.25	63.38	23.61	− 0.09
Es.Ar	13.64	141.09	44.17	21.37	0.16
Ct.Ar/Tt.Ar	0.091	0.596	0.322	0.12	− 0.55***
On.Ar	0.015	0.052	0.032	0.01	− 0.64***
On.Pm	0.433	0.831	0.632	0.1	− 0.67***
On.Cr	0.858	0.945	0.91	0.02	0.67***

*SD* standard deviation, *r* Pearson’s correlation, *rho* Spearman’s correlation (in italics)

*p-value < 0.05; ***p-value < 0.001.

### Differences between population samples and sexes

The histomorphometric parameters were explored using Pearson´s correlation coefficients or Spearman’s Rank correlation for each subgroup (sex and population) to assess the strength and direction of the relationship between known age and each variable (Table 4). A similar pattern in the direction of the association (positive or negative) as seen for the total sample was observed on most of the variables for the four subgroups. For both sex and population samples, the overall highest correlations were observed for OPD-related variables, Ct.Ar and Ct.Ar/Tt.Ar, and osteon metric parameters (*see supplementary material for sex and population subgroup correlations linear relationship and density plots*).

**Table 4:** Pearson’s correlation and Spearman’s rank correlation for the raw and composite histomorphometric parameters and age for the two sub-samples: population and sex

	Sex		Population sample	
	Male	Female	Crete	Greek-Cyprus
N.On	− 0.11	− 0.36**	− 0.27	− 0.34*
N.On.Fg	0.44**	0. 29*	0.31*	0.31*
N.On.Tt	0.11	− 0.11	− 0.05	− 0.11
OPD(I)	0.51**	0.39**	0.42**	0.43**
OPD(F)	0.79**	0.82**	0.77**	0.81**
OPD	0.79**	0.69**	0.68**	0.73**
Ct.Ar	− 0.42**	− 0.73**	− 0.68**	− 0.58**
Tt.Ar	− 0.11	− *0.07*	− *0.30*	− 0.08
Es.Ar	*0.08*	0.17	− *0.10*	0.23
Ct.Ar/Tt.Ar	− 0.27	− 0.70**	− 0.43**	− 0.72**
On.Ar	− 0.54**	− 0.69**	− 0.66**	− 0.73**
On.Pm	− 0.58**	− 0.77**	− 0.69**	− 0.76**
On.Cr	0.67**	0.68**	0.68**	0.64**

Italics indicates Spearman´s rank correlation

*p-value < 0.05, **p-value< 0.01

Age distribution for the sex and population samples was examined confirming approximately normality (*p* > 0.05). The comparison of age distributions between the sexes and the populations demonstrated non statistically significant results (*t*[86) =  − 0.109, *p* = 0.91 and *t*(86) =  − 1.364, *p* = 0.17, respectively). The differences between the sexes and populations were further explored to understand whether the histomorphometric parameters differ between each group in relation to age. Four variables indicated statistically significant differences between the sexes. N.On.Tt presented a different distribution between the sexes (*t*(81.38) = 6.09, *p* = 0.02) with higher values for males than for females (312 and 257 total osteon means, respectively). N.On, Tt.Ar, and Es.Ar presented significantly higher values for males than for females (*U* = 648, *z* =  − 2.61, *p* = 0.009; *U* = 499, *z* =  − 3.86, *p* = 0.001; and *U* = 546, *z* = -3.47, *p* = 0.001, respectively). Regarding population sample differences, three variables were different between the Cretans and Greek-Cypriots with values being consistently higher for Greek-Cypriots when compared to Cretans (N.On = *t*(83.82) = 8.27, *p* = 0.005; N.On.Tt = (*t*(83.52) = 16.76, *p* < 0.001), and OPD(F) = (*t*(83.26) = 4.25, *p* = 0.042). N.On.Fg differed statistically significantly between the samples showing again higher values for the Greek-Cypriots (*U* = 440, *z* =  − 4.38, *p* < 0.001).

Sex and population sample effect was further assessed using one-way ANCOVA. For all the dependent variables tested, known age was reported to be a statistically significant covariate. Only those parameters that do not violate the assumption of homogeneity of regression slopes are presented here. Regarding sex differences and the parameters explored, the only variable indicating a statistically significant sex effect was Ct.Ar (LnCort.Ar: *F*(1, 85) = 7.36, *p* < 0.010, partial *η*^2^ = 0.080). Post hoc analysis with Bonferroni adjustment indicated that Ct.Ar was higher in males than in females with a mean difference of 3.46 (95% CI, 0.713–6.219, *p* < 0.010), with bootstrapping confirming the reported results. In relation to population sample effect, Ct.Ar, On.Ar, and On.Pm demonstrated statistically significant differences between Cretans and Greek-Cypriots. After adjustment of age, sample effect was found statistically significant for Ct.Ar (LnCt.Ar: *F*(1,85) = 12.15, *p* < 0.010, partial *η*^2^ = 0.125). Post hoc analysis was carried out with Bonferroni adjustment showing that the mean difference was statistically significant with Greek-Cypriots reporting 4.16 larger Ct.Ar than the Cretans, being further confirmed by the bootstrapping procedure (95% CI: 1.429 to 6.901, *p* < 0.001). When age was adjusted as a covariate, the sample effect was statistically significant for On.Ar and On.Pm (*F*(1,85) = 9.996, *p* < 0.010, partial *η*^2^ = 0.105, and *F*(1,85) = 9.992, *p* < 0.010, partial *η*^2^ = 0.106, respectively). Post hoc test with Bonferroni adjustment indicated that the Greek-Cypriot sample has a greater On.Ar than the Cretan sample with a significant mean difference of 0.005 (95% CI: 0.002 to 0.008, *p* < 0.01), and a higher value for On.Pm than the Cretan sample with a mean difference of 0.05 (95% CI: 0.019 to 0.082, *p* < 0.01).

### GLM: population-specific standards for age estimation

Simple and multiple GLM were generated using the entire sample through the inclusion of those variables that demonstrated a statistically significant relationship with age (Table 3). Only those models meeting the assumptions for linear regression, producing a SEE lower than 15 years, the lowest AIC and BIC values as well as the optimal LOO results are reported. Other considerations related to the general applicability of the models such as repeatability and reproducibility of the histomorphometric parameters were also taken into account. Therefore, only those GLM meeting the above criteria will be fully presented and described (Table 5). For sex and population sample-specific models, only those models showing an improvement in accuracy and fitness indicators as compared to the total sample models will be provided.

**Table 5:** Summary of total sample, sex and population-specific models with fitness indicators and cross-validation results

	M1 total	M2 total	M3 total	M4 total	M5 total	M1 males	M1 females	M2 females	M1 Greek-Cypriots	M2 Greek-Cypriots	M3 Greek-Cypriots	M4 Greek-Cypriots
OPD	2.893^***^		1.982^***^	1.577^***^		2.040^***^			2.549^***^		1.507^***^	
On.Pm		− 115.240^***^		− 48.903^***^			− 74.338^***^			− 113.681***	− 73.831^***^	− 86.248^***^
On.Cr			334.384^***^	241.798^***^	454.948^***^	302.931^***^		399.886^***^				
Ct.Ar					− 0.866^***^		− 1.220^***^	− 1.453^***^				
Ct.Ar./Tt.Ar												− 40.518^***^
Constant	15.660^***^	133.224^***^	− 274.591^***^	− 153.141^**^	− 337.125^***^	− 247.983^***^	128.742^***^	− 276.939^***^	21.361^***^	136.234***	85.982^***^	131.744^***^
*Model summary*												
Observations	88	88	88	88	88	40	48	48	47	47	47	47
*R*^2^	0.495	0.448	0.607	0.654	0.58	0.628	0.644	0.696	0.539	0.572	0.691	0.644
AIC	702.189	710.038	682.054	672.968	687.905	305.326	376.898	369.218	351.384	347.879	334.66	341.257
BIC	709.621	717.47	691.963	685.354	697.814	312.082	384.383	376.703	356.934	353.43	342.059	348.657
*F* statistic	84.289^***^	69.758^***^	65.723^***^	52.872^***^	58.761^***^	31.263^***^	40.671^***^	51.632^***^	52.666^***^	60.23***	49.118^***^	39.804^***^
Linear regression diagnostics												
Adjusted *R*^2^	0.489	0.441	0.598	0.641	0.57	0.608	0.628	0.683	0.529	0.563	0.677	0.628
MAE	9.223	10.192	8.389	7.938	8.823	8.822	7.455	8.225	6.906	7.07	5.889	6.313
SEE	12.784	13.367	11.339	10.71	11.722	10.346	11.658	10.762	9.748	9.392	8.078	8.665
Cross-validated (LOO) diagnostics												
Adjusted *R*^2^	0.475	0.421	0.581	0.617	0.552	0.566	0.595	0.655	0.494	0.493	0.639	0.587
MAE	9.427	10.444	8.691	8.348	9.135	8.103	9.654	8.763	7.25	7.25	6.336	6.798
SEE	12.888	13.535	11.513	11.015	11.905	10.769	12.045	11.115	10.007	10.007	8.455	9.053

**p-value < 0.01; ***p-value < 0.001.

The total sample dataset (*N* = 88) was used to conduct simple linear regression analysis through the inclusion of single raw or composite parameters producing models providing a SEE ranging from a maximum of 17.49 years for N.On and a minimum of 11.32 for OPD(F). Although the latter model provided the lowest SEE after cross-validation, this model was not selected as optimal due to the inter-observer error reported for this specific parameter somewhere else [9, 12, 30]. The analysis results in M1 as the optimal univariate model, which includes OPD as single parameter producing a SEE of 12.78 years and cross-validated SEE of 12.88 years (Adj. *R*^2^ = 0.48). For the application of the model for age estimation on an unknown individual, the practitioner needs to apply the values reported in Table 5. For example, the value collected for OPD needs to be multiplied by its unstandardised coefficient (2.893) and the constant added (15.660). The resulting value will be the age estimate (age estimate = (2.893*OPD value) + 15.660). The next best model (M2) includes On.Pm producing a slightly higher SEE and cross-validated SEE (13.36 and 13.53, respectively) (Table 5). Multiple linear regression analysis was performed exploring all possible combination of parameters having into account the considerations presented above. Three models are provided which include a combination of OPD and osteon metrics parameters (M3 and M4 Total) as well as a model that does not require the inclusion of OPD, but instead selects On.Cr and Ct.Ar (M5 Total). Among these three models, the lowest SEE (10.71 years) and cross-validated SEE (11.01 years) are reported for M4 Total (Adj. *R*^2^ = 0.64).

Sex-specific models were generated by dividing the dataset into males and females, separately, and running simple and multiple linear regression analysis (Table 5). The optimal model for males (M1 Males) produces a SEE and cross-validated SEE of 10.35 and 10.76 years by the inclusion of OPD and On.Cr. The optimal model produced for females incorporated On.Cr and Ct.Ar with adjusted *R*^2^ of 0.68 and SEE and cross-validated SEE of 10.76 and 11.11 years, respectively.

**Fig. 4:**
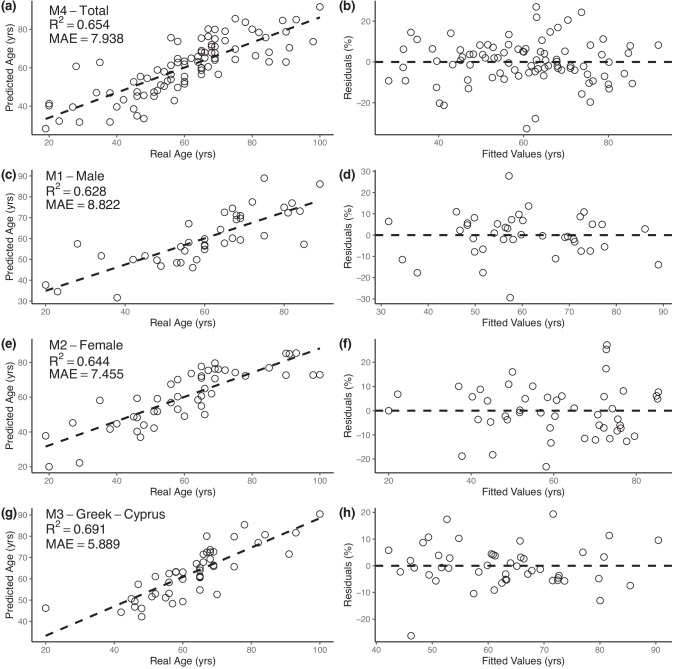
Diagnostic plots for the relationship between known age and predicted age, and the fitted values and residuals for the best models for each dataset: **a**–**b** M4 total sample; **c**–**d** M1 males; **e**–**f** M2 females; **g**–**h** M3 Greek-Cypriots

None of the population-specific models for Cretans showed an improvement when compared to the total sample models, and thus, no results are reported here. However, the Greek-Cypriots dataset (*N* = 47) produced four optimal models (Table 5). A combination of OPD and On.Pm (M3 Greek-Cypriots) and On.Pm and Ct.Ar/Tt.Ar (M4 Greek-Cypriots) resulted in SEE and cross-validated SEE around 8 years and adjusted *R*^2^ of 0.67 and 0.63, respectively. Figure 4 presents the diagnostic plots for the best models for the total sample, and for the sex and population-specific subsamples.

## Discussion

It is known that the relationship between micromorphological features and age is not fully consistent, following the same pattern as other biological age markers [34]. Thus, intra- and inter-variability due to sex, pathology, nutrition, physical activity, and genetics – among other factors — may consequently affect not only the application of the methods but also the expression of the microstructural features [35]. Information regarding life style or pathology is not available for the sample under study, and it will be recommended for future research performed on Mediterranean populations to reach a full understanding of remodelling rates in relation to biomechanics and the impact of specific disorders [36, 37].

There is still an open debate regarding sex differences in histomorphometric variables, with some studies reporting sex differences in osteonal variables [6, 38] while others did not report any [1, 39]. Whether the earlier completion of the cortex in females would have an impact on the histological variables and on the age estimates due to differences in the mean tissue age needs further investigation [35]. In the early years of adulthood, males present around 40% larger bone area than females. This observation is associated with the larger body size of males. Additionally, a decrease in cortical area produced at the cross-section periosteal and endosteal regions observed for both sexes around middle age would potentially contribute to these differences [40]. As humans age, males and females experience a general decline in bone formation at the periosteal surface with a reduction in bone formation and continued resorption at the basic multicellular unit level of bone, resulting in a negative remodelling balance [41]. This imbalance is triggered by an increase in cortical remodelling which accelerates cortical and trabecular thinning with interstitial bone becoming highly mineralised in postmenopausal women [41]. Considering that 73% of the females in this sample are over 50 years old, the differences observed on the histomorphometric parameters may result from postmenopausal associated osteoporosis and related bone loss. The sex differences reported here are in agreement with other studies [38, 42, 43], although caution with microstructures, definition and skeletal element used by the other authors must be considered. In our results, Ct.Ar showed statistically significant differences with age as a covariate, and Tt.Ar and Es.Ar reported differences when the two sexes were directly compared, in agreement with other studies [42, 43]. Perhaps, the effect of periosteal apposition seen in postmenopausal women as an adaptive response to the decrease of cortical bone strength and to the increase in bone fragility is responsible for the observed sex differences. However, this can be simply a reflection of sexual dimorphism in rib size as other studies stated substantial sexual differences for Cretans and Greek-Cypriots [17, 18]. The key parameter used for age estimation, OPD, did not show any statistically significant differences between the sexes in disagreement with other studies [42]. Further research should be performed on a larger sample with a higher representation of individuals from different age ranges, overcoming the limitations of the current sample and further confirming our results.

Differences between the two population samples, Cretans and Greek-Cypriots, should be minimal. Both share dietary and cultural habits, along with similar climate [18]. However, the populations overall health and habits could have been impacted by differences in historical events. For example, the invasion of Cyprus in 1975 increased the poverty levels for Greek-Cypriots [44] and both islands experienced shifts in economic activities [45], (10.1080/13683500308667943).  Frequency number of osteons and OPD(F) showed statistically significant differences with higher values for Greek-Cypriots in comparison to Cretans. The same pattern is seen for Ct.Ar, On.Ar, and On.Pm with age as a covariate. This could mean that the packing factor effect relating osteons number, osteon size, and cortex dimensions may be reflected in the trend observed for OPD values between the samples (mean OPDs: Cretans = 14.50, Greek-Cypriots = 16.30) [46]. Cortical bone phenotypic traits — e.g. cross-sectional area and density — are determined in the early years of life. However, during growth and development, these traits are also modulated by environmental factors, with disease and life-style influencing the amount of bone mass reached later in life [47]. Moreover, polygenetic interactions and aging effects on the molecular and cellular processes, decrease of muscle mass, and life style — among other factors — are responsible of inter-individual and inter-population bone modelling and remodelling variability [48]. This topic deserves further investigation, and the inclusion of other parameters such as bone mineral density or other bone surfaces such as trabecular tissue might assist in future research related to the populations under study.

The ultimate goal of this research was the generation of rib histological age estimation standards, as it was demonstrated that existing methods presented several drawbacks for the sample under study [12]. Twelve GLM were reported as the optimal ones considering prediction accuracy, goodness of fit indicators, and cross-validation (Table 5), as well as observer errors [9, 30]. If the sex of the individual is known, the use of sex-specific prediction equations is recommended, since overall, they provide slightly more accurate results than the general equations. Regarding the application of the models and considering the potential fragmentation of the remains, sex might be unknown implying that equations for the total sample should be applied. Within those, M3 to M5 provided similar accuracy rates, with the optimal model being M4 included OPD, On.Pm, and On.Cr. Although all these variables reported overall acceptable observer errors [5, 12, 49], the practitioner must have a suitable training on histomorphometric assessment to ensure reliable and accurate results, especially when OPD is assessed. Metric measurements on secondary osteons might be more feasible for experts with basic training in histology, and thus, M2 and M5 including just On.Pm and On.Pm and Ct.Ar, respectively, might be a better option. Note that On.Cr observer error agreement ranged from poor to good in several studies [12, 13], and standardisation for this parameter might need further attention in the future [49]. Regarding sample-specific models, the total GLM should be applied if the unknown individual is suspected to be from Crete as the Cretan dataset did not produce lower errors than the total sample dataset. Now, for individuals of Greek-Cypriot origin, the SEE ranged from 8 to 9.75 years accounting for the lowest errors reported for all the tested models. The same principle applies here for the use of M2 and M4 to avoid errors in the assessment of secondary osteon densities recorded by inexperienced anthropologist, as they apply histological methods for assessing AAD on human skeletal remains.

In general, most of the predicting models included osteon density and/or osteon metrics as age predictors confirming their use for histological aging formulae. Even if OPD is still one of the best predictors, the inclusion of osteon size and shape descriptors has been recommended by other authors; osteon metric parameters are not directly subject to the asymptotic effect, and thus, their use compensates the inconsistencies of OPD for advanced age samples [5]. It is expected that the older the sample, the higher the error rates. This is due to a phenomenon known as the “trajectory effect” which implies that biomechanical and physiological changes in the aging indicators undergo more alterations as the later years in the life span are approaching [50]. Although individuals from younger cohorts should be included in future research in order to fully represent the general population, life expectancy in these countries is increasing steadily, and thus, development of anthropological methods for the elderly are currently required and methods validation needed [51, 52]. Our method falls within the expectations reported for histological methods, demonstrating their suitability for age estimation in adults from modern human remains.

## Conclusions

The present research is a continuation of the validation study conducted on the Mediterranean sample under study that highlighted the potential need to explore remodelling rates and histomorphometric data on Cretans and Greek-Cypriots. Our results demonstrate that several parameters differ between the sexes and the populations, possibly accounting for variation related to aging as a natural process and age-related changes such as hormonal alterations. Other considerations such as life history and clinical data would be needed to provide further conclusions on the sample under study. The proposed aging equations can be applied on unknown individuals in future forensic cases in Crete and in the Republic of Cyprus. Especially for Greek-Cypriots, this method could potentially assist in the identification of more than a thousand individuals that have yet to be identified, according to the Committee of Missing Persons (www.cmp-cyprus.org). Further research will focus on examining a larger sample size including a detailed representation of adult age cohorts, as well as validating the presented age estimation prediction equations on individuals or collections of the same populations.

## Supplementary Information

Below is the link to the electronic supplementary material.

Supplementary file1 (DOCX 1187 KB)
