# Supplementary material for: Age estimation for two Mediterranean populations: rib histomorphometry applied to forensic identification and bone remodelling research
Source: Int J Legal Med. 2022 Apr 8;136(5):1469–81. doi: 10.1007/s00414-022-02812-2 (PMC9375747; doi:10.1007/s00414-022-02812-2)

**SUPPLEMENTARY MATERIAL**

Descriptive statistics for the entire sample divided by sex subsamples.

|  | **MALE** N=40 | Descriptive statistics | | | | |
| --- | --- | --- | --- | --- | --- | --- |
|  | **FEMALE** N=48 | Min | Max | Mean | SE | SD |
| VARIABLES | Known Age | 20 | 89 | 60.1 | 2.61 | 16.53 |
|  |  | 19 | 100 | 60.52 | 2.76 | 19.11 |
|  | N.On | 66 | 399 | 194.8 | 11.78 | 74.52 |
|  |  | 46 | 355 | 154.54 | 9.83 | 68.09 |
|  | N.On.Fg | 47 | 212 | 118 | 7.18 | 45.44 |
|  |  | 23 | 224 | 102.65 | 6.58 | 45.58 |
|  | N.On.Tt | 142 | 583 | 312.8 | 17.05 | 107.8 |
|  |  | 96 | 507 | 257.19 | 14.74 | 102.1 |
|  | OPD(I) | 5.38 | 13.04 | 9.38 | 0.32 | 2.02 |
|  |  | 3.56 | 13.72 | 8.98 | 0.35 | 2.4 |
|  | OPD(F) | 2.18 | 12.85 | 6.1 | 0.42 | 2.66 |
|  |  | 0.93 | 12.64 | 6.43 | 0.4 | 2.74 |
|  | OPD | 7.65 | 24.93 | 15.48 | 0.65 | 4.08 |
|  |  | 4.49 | 25.62 | 15.42 | 0.66 | 4.61 |
|  | Ct.Ar | 9.26 | 44.77 | 21.16 | 1.37 | 8.64 |
|  |  | 6.38 | 39.95 | 17.59 | 1.02 | 7.07 |
|  | Tt.Ar | 26.82 | 155.25 | 74.66 | 4.24 | 26.79 |
|  |  | 32.65 | 90.6 | 53.98 | 2.23 | 15.42 |
|  | Es.Ar | 14.52 | 141.09 | 53.5 | 3.97 | 25.12 |
|  |  | 13.64 | 72.87 | 36.39 | 1.97 | 13.64 |
|  | Ct.Ar/Tt.Ar | 0.091 | 0.572 | 0.304 | 0.02 | 0.12 |
|  |  | 0.152 | 0.596 | 0.336 | 0.02 | 0.13 |
|  | On.Ar | 0.016 | 0.052 | 0.032 | 0.001 | 0.01 |
|  |  | 0.015 | 0.051 | 0.031 | 0.001 | 0.01 |
|  | On.Pm | 0.446 | 0.831 | 0.636 | 0.02 | 0.1 |
|  |  | 0.433 | 0.818 | 0.629 | 0.02 | 0.11 |
|  | On.Cr | 0.858 | 0.945 | 0.913 | 0.003 | 0.02 |
|  |  | 0.859 | 0.942 | 0.908 | 0.002 | 0.02 |

Descriptive statistics for the entire sample divided by population subsamples.

|  | **CRETE** N=41 | Statistics | | | | |
| --- | --- | --- | --- | --- | --- | --- |
|  | **CYPRUS** N=47 | Min | Max | Mean | SE | SD |
| **VARIABLES** | Known Age | 19 | 98 | 57.49 | 3.31 | 21.17 |
|  |  | 20 | 100 | 62.81 | 2.07 | 14.2 |
|  | N.On | 60 | 299 | 150.1 | 9.18 | 58.81 |
|  |  | 46 | 399 | 192.68 | 11.62 | 79.64 |
|  | N.On.Fg | 23 | 183 | 86.83 | 5.24 | 33.58 |
|  |  | 50 | 224 | 129.51 | 6.74 | 46.23 |
|  | N.On.Tt | 111 | 482 | 236.93 | 12.82 | 82.11 |
|  |  | 96 | 583 | 322.19 | 16.41 | 112.51 |
|  | OPD(I) | 3.56 | 13.04 | 8.85 | 0.36 | 2.33 |
|  |  | 4.48 | 13.72 | 9.44 | 0.31 | 2.14 |
|  | OPD(F) | 0.93 | 11.88 | 5.66 | 0.42 | 2.71 |
|  |  | 2.25 | 12.85 | 6.83 | 0.38 | 2.59 |
|  | OPD | 4.49 | 24.93 | 14.5 | 0.7 | 4.5 |
|  |  | 8.03 | 25.62 | 16.26 | 0.6 | 4.09 |
|  | Ct.Ar | 8.17 | 42.11 | 17.74 | 1.19 | 7.63 |
|  |  | 6.38 | 44.77 | 20.49 | 1.19 | 8.13 |
|  | Tt.Ar | 26.82 | 155.25 | 60.29 | 3.74 | 23.96 |
|  |  | 33.5 | 115.65 | 66.08 | 3.39 | 23.23 |
|  | Es.Ar | 13.64 | 141.09 | 42.55 | 3.61 | 23.12 |
|  |  | 15.07 | 87.79 | 45.58 | 2.9 | 19.87 |
|  | Ct.Ar/Tt.Ar | 0.091 | 0.596 | 0.316 | 0.02 | 0.13 |
|  |  | 0.156 | 0.556 | 0.326 | 0.02 | 0.11 |
|  | On.Ar | 0.016 | 0.05 | 0.03 | 0.001 | 0.01 |
|  |  | 0.015 | 0.052 | 0.033 | 0.001 | 0.01 |
|  | On.Pm | 0.446 | 0.809 | 0.622 | 0.02 | 0.11 |
|  |  | 0.433 | 0.831 | 0.646 | 0.01 | 0.09 |
|  | On.Cr | 0.858 | 0.942 | 0.905 | 0.003 | 0.02 |
|  |  | 0.859 | 0.945 | 0.913 | 0.002 | 0.02 |

Histological parameters and age presenting Pearson’s correlation coefficient, linear relatioship and density plots for males and females.


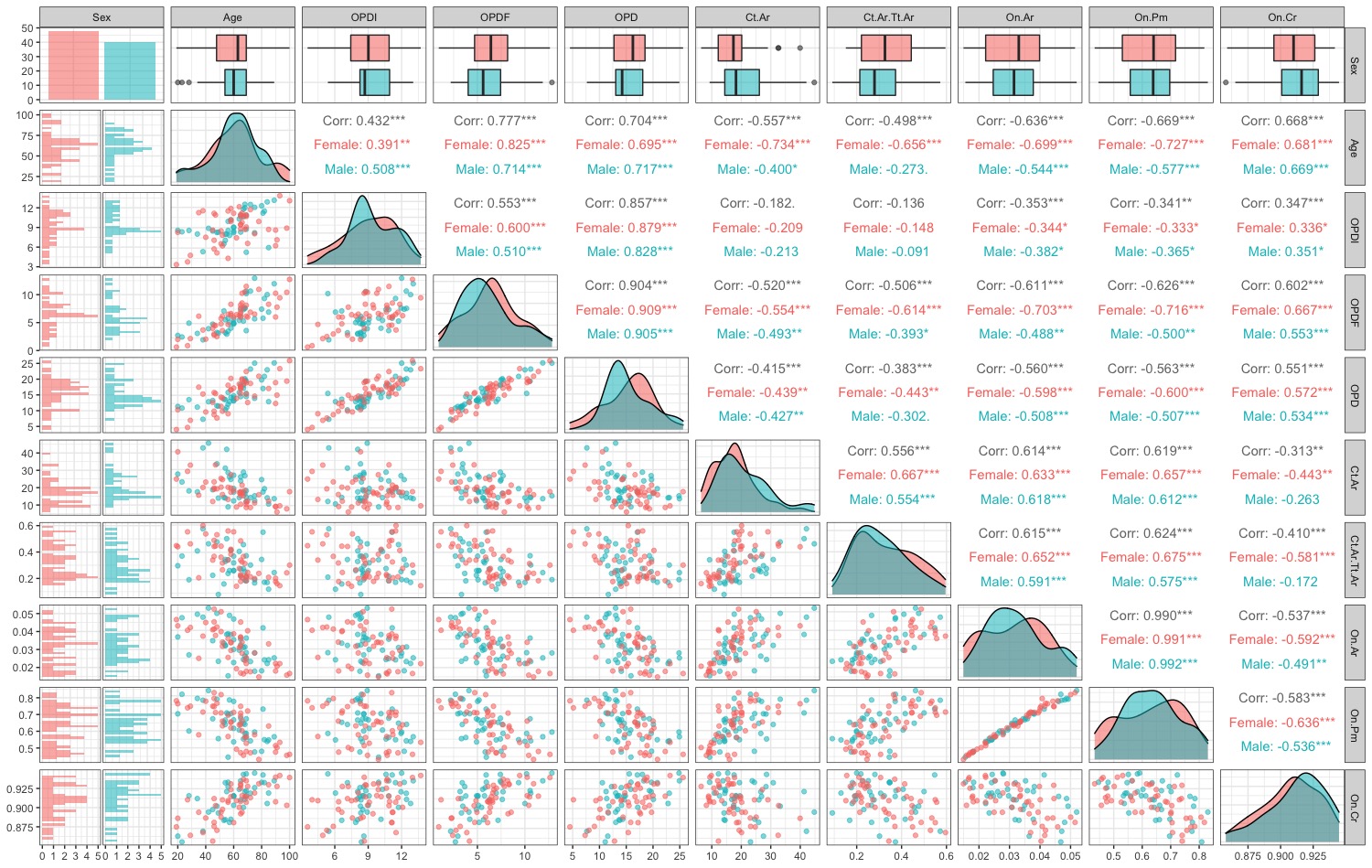


Histological parametrs and age presenting Pearson’s correlation coefficient, linear relatioship and density plots for Cretans and Greek-Cypriots.


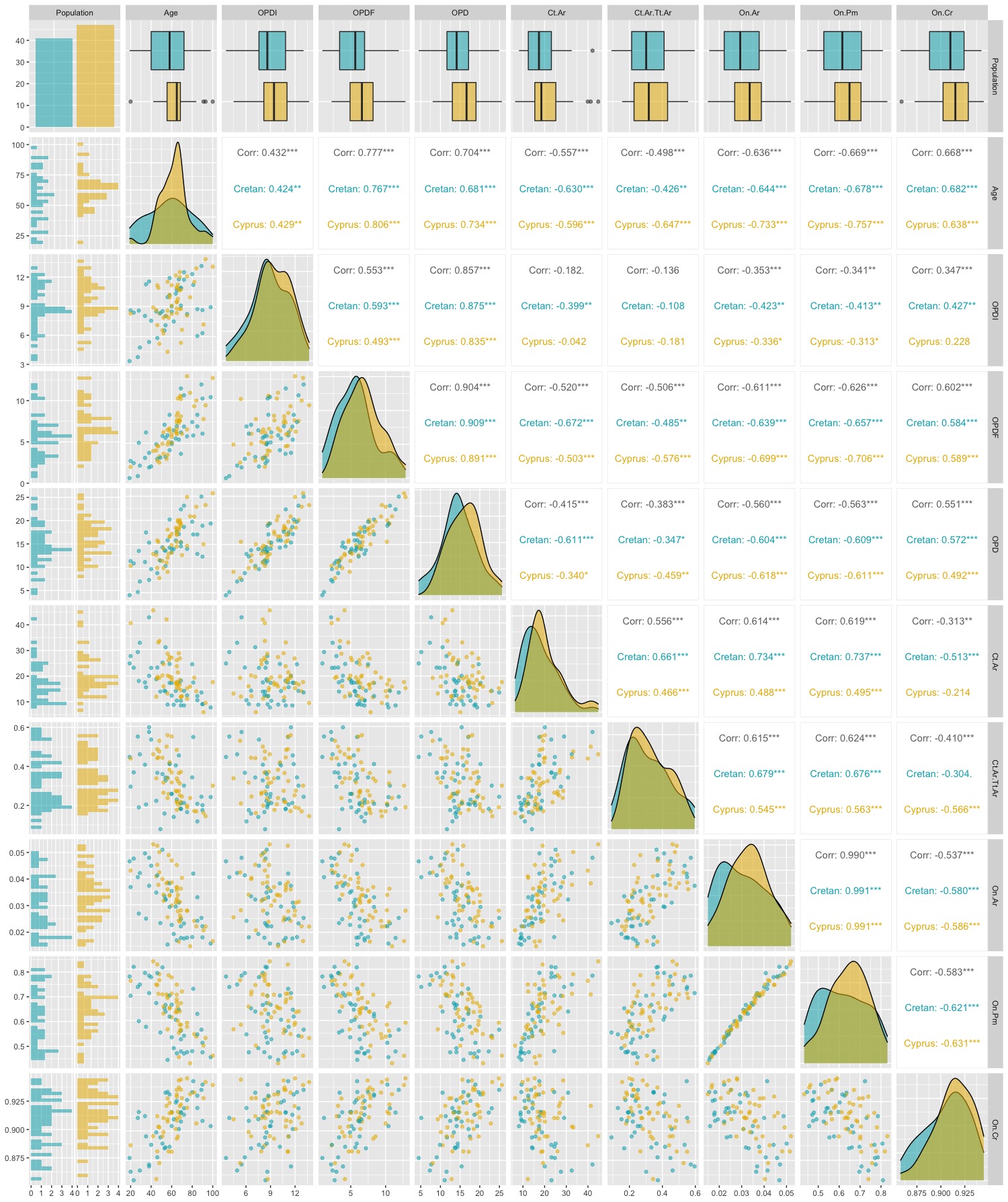

Supplement: Supplementary file 1 — Supplementary file1 (DOCX 1187 KB) [file 414_2022_2812_MOESM1_ESM.docx]
